# Case Report: Dual toxicity of voriconazole in a CYP2C19 poor metabolizer: a case analysis of life-threatening neutropenia and hepatocellular injury

**DOI:** 10.3389/fphar.2026.1875506

**Published:** 2026-08-21

**Authors:** Ke-Ke Liu, Wei-Jing Gong

**Affiliations:** 1 Department of Pharmacy, Huaihe Hospital of Henan University, Kaifeng, Henan, China; 2 Department of Pharmacy, Union Hospital Tongji Medical College Huazhong University of Science and Technology, Wuhan, Hubei, China

**Keywords:** CYP2C19, drug-induced liver injury, neutropenia, poor metabolizer, voriconazole

## Abstract

**Background:**

Voriconazole, a first-line therapy for invasive aspergillosis, exhibits variable pharmacokinetics due to *CYP2C19* polymorphisms. However, severe hematological toxicity, particularly life-threatening neutropenia, remains under-recognized. The concomitant occurrence of dual severe organ toxicities in CYP2C19 poor metabolizers (PMs) poses a significant clinical challenge.

**Case:**

We report a case of a 71-year-old Chinese male with invasive pulmonary aspergillosis. After approximately 40 days of oral voriconazole therapy (400 mg q12h loading dose, then 200 mg q12h maintenance), he sequentially developed grade 4 neutropenia (Absolute Neutrophil Count nadir 0.02 × 10^9^/L) with fever and grade 3 hepatocellular liver injury (Alanine Aminotransferase [ALT] peak 738 U/L; R ratio approximately 65.9). Pharmacogenomic testing performed during hospitalization on 27 March 2025 revealed a CYP2C19 *2/*3 PM genotype. Causality assessments using the Naranjo and RUCAM scales rated both events as probable in relation to voriconazole. Following discontinuation of voriconazole, antifungal therapy was sequentially changed to isavuconazonium sulfate, nebulized amphotericin B liposome, and intravenous amphotericin B cholesteryl sulfate complex, together with granulocyte colony-stimulating factor and hepatoprotective therapy. The patient’s neutrophil count and liver function improved, and he was discharged in stable condition with isavuconazonium sulfate capsules 200 mg qd after discharge. After discharge, he reported feeling well but did not undergo follow-up laboratory or imaging examinations.

**Conclusion:**

This case illustrates a clinical scenario in which pre-emptive pharmacogenomic testing, vigilant monitoring of complete blood counts alongside liver function, and integration with therapeutic drug monitoring (TDM) may enhance safety in patients requiring long-term voriconazole therapy. The absence of TDM data precludes definitive confirmation of voriconazole overexposure; nevertheless, the convergence of *CYP2C19* PM genotype, temporal relationship, dechallenge, and structured causality assessment renders presumed drug accumulation the most plausible explanation for the observed toxicities while not excluding all competing contributors.

## Introduction

Voriconazole, a second-generation broad-spectrum triazole antifungal, remains a cornerstone in the treatment of invasive aspergillosis and other serious fungal infections due to its potent activity (Patterson et al., 2016; Douglas et al., 2021; Epelbaum et al., 2024). However, its clinical utility is significantly constrained by pronounced inter- and intra-individual pharmacokinetic variability, attributable to nonlinear metabolism primarily mediated by the CYP2C19 enzyme (Chen et al., 2018). This variability translates into unpredictable drug exposure, posing challenges in balancing therapeutic efficacy against the risk of concentration-dependent adverse effects.

Therapeutic Drug Monitoring (TDM) of voriconazole trough concentrations has become a standard of care to mitigate toxicity, with hepatotoxicity being the most widely recognized and monitored adverse event (Ma et al., 2023). In contrast, voriconazole-induced hematological toxicity, particularly severe and life-threatening neutropenia or agranulocytosis, receives considerably less attention despite being listed in the prescribing information. The concomitant occurrence of both severe hematological and hepatic toxicity in a single patient is exceptionally rare and poorly characterized in the literature.

*CYP2C19* gene polymorphisms are the principal genetic determinant of voriconazole metabolism. Individuals carrying two loss-of-function alleles (e.g., *2, *3) are classified as poor metabolizers (PMs), a phenotype prevalent in approximately 14.7% of Asian populations (Du et al., 2024; Katada et al., 2025). PMs exhibit substantially reduced drug clearance, leading to elevated and sustained voriconazole plasma concentrations, which have been correlated with an increased incidence of overall adverse reactions, including liver injury (Gu et al., 2023; Blanco-Dorado et al., 2020). However, the specific risk and mechanistic pathway for severe neutropenia in this pharmacogenetic context remain largely unexplored.

This case report details a life-threatening dual toxicity--grade 4 neutropenia with concomitant grade 3 Drug-Induced Liver Injury (DILI)--in a CYP2C19 PM patient receiving oral voriconazole therapy. By integrating clinical phenotyping with pharmacogenetic analysis and structured causality assessment, we aim to: 1) describe the presentation and temporal sequence of this dual toxicity, 2) explore the possible contribution of CYP2C19 PM status, 3) discuss the complex interplay among myelosuppression, infection, concomitant medications, and antifungal switching, and 4) propose a cautious risk-mitigation strategy for voriconazole use in high-risk populations.

## Case presentation

A 71-year-old male (height 167 cm; body weight 60 kg in February 2025 and 55 kg on 24 March 2025; admission BMI 19.72 kg/m^2^) was admitted on 24 March 2025, for recurrent fever for 7 days, with a history of cough and sputum for over 1 month. His past history included laparoscopic excision of a left peripelvic renal cyst on 19 July 2022. His past medical history was negative for diabetes mellitus, hypertension, chronic liver disease, and other chronic comorbidities. He had no long-term medication before admission, had a history of smoking, and had a history of alcohol consumption of approximately 50 mL of liquor per day, but had abstained since early February 2025. Nutritional assessment revealed a body mass index of 19.72 kg/m^2^ (underweight), weight loss from 60 kg (February 2025) to 55 kg (March 2025), and a serum albumin decline from 31 g/L at baseline to 26.6 g/L at admission, indicating a moderate malnutrition risk. In early February 2025, his chest CT from another hospital revealed bilateral pulmonary infectious lesions. Pathogen-targeted next-generation sequencing of bronchoalveolar lavage fluid identified *Aspergillus flavus* (3,295 reads) and *Aspergillus fumigatus* (118 reads). Based on clinical presentation, radiological findings, and microbiological evidence, a diagnosis of probable invasive pulmonary aspergillosis was established. Oral voriconazole therapy was initiated on 9 February 2025 (loading dose 400 mg q12h for 1 day, then maintenance dose 200 mg q12h), alongside ceftizoxime and moxifloxacin. His condition improved, and he was discharged on February 16, continuing voriconazole as outpatient therapy. According to infectious disease guidelines for invasive aspergillosis, a minimum treatment duration of 6–12 weeks is recommended. Therefore, the planned course was for chronic therapy.

Before voriconazole initiation, laboratory results were as follows (Table 1): White Blood Cell Count (WBC) 6.31 × 10^9^/L, Absolute Neutrophil Count (ANC) 5.47 × 10^9^/L, Hemoglobin (Hb) 151 g/L, Platelet (PLT) count 101 × 10^9^/L, Eosinophils (EOS) count 0.05 × 10^9^/L, Alanine Aminotransferase (ALT) 35 U/L, Aspartate Aminotransferase (AST) 89 U/L, Gamma-Glutamyl Transferase (GGT) 40 U/L, Total Bilirubin (TBil) 9.56 umol/L, Direct Bilirubin (DBil) 3.64 umol/L, Albumin (Alb) 31 g/L, International Normalized Ratio (INR) 1.0, Serum Creatinine (Scr) 99 umol/L, Blood Urea Nitrogen (BUN) 11.2 mmol/L, estimated Glomerular Filtration Rate (eGFR) 71.4 mL/min/1.73 m^2^, C-Reactive Protein (CRP) 150.7 mg/L, and Procalcitonin (PCT) 5.38 ng/mL. At voriconazole initiation, WBC was 4.98 × 10^9^/L, ANC 3.93 × 10^9^/L, ALT 62 U/L, AST 44 U/L, GGT 38 U/L, TBil 16.46 umol/L, DBil 6.95 umol/L, Alb 28 g/L, and INR 1.2. Baseline Alkaline Phosphatase (ALP) values before and at the start of voriconazole were not available.

**TABLE 1:** Key laboratory values before voriconazole, at voriconazole initiation, at ALT peak, and at discharge.

Parameter	Before voriconazole	At voriconazole initiation	ALT peak	Discharge
WBC (× 10^9^/L)	6.31	4.98	1.51	20.43
ANC (× 10^9^/L)	5.47	3.93	0.87	14.74
Hb (g/L)	151	111	116	114
PLT (× 10^9^/L)	101	101	250	271
EOS (× 10^9^/L)	0.05	0.06	0.01	0.07
ALT (U/L)	35	62	738	123
AST (U/L)	89	44	369	84
ALP (U/L)	NA	NA	42	41
GGT (U/L)	40	38	138	194
TBil (µmol/L)	9.56	16.46	16.6	8.2
DBil (µmol/L)	3.64	6.95	11.3	3.8
Alb (g/L)	31	28	26.6	28.1
INR	1.0	1.2	1.1	1.01
Scr (µmol/L)	99	69	88.9	72.4
BUN (mmol/L)	11.2	7.1	7.57	8.5
eGFR (mL/min/1.73 m^2^)	71.4	111.07	74.87	89.01
CRP (mg/L)	150.7	108.93	143.35	14.1
PCT (ng/mL)	5.38	0.76	NA	0.08

WBC, white blood cell; ANC, absolute neutrophil count; Hb, hemoglobin; PLT, platelet; EOS, eosinophils; ALT, alanine aminotransferase; AST, aspartate aminotransferase; ALP, alkaline phosphatase; GGT, gamma-glutamyl transferase; TBil, total bilirubin; DBil, direct bilirubin; Alb, albumin; INR, international normalized ratio; Scr, serum creatinine; BUN, blood urea nitrogen; eGFR, estimated glomerular filtration rate; CRP, C-reactive protein; PCT, procalcitonin.

Approximately 30 days into voriconazole therapy (March 12), a Complete Blood Count (CBC) first revealed neutropenia (ANC 1.35 × 10^9^/L). On day 36 (March 17), the patient developed fever. By day 39 (March 20), CBC demonstrated severe neutropenia (WBC 0.91 × 10^9^/L, ANC 0.02 × 10^9^/L). Voriconazole was discontinued at the referring hospital, and ceftazidime plus Granulocyte Colony-Stimulating Factor (G-CSF) was initiated, with poor response. Persistent fever prompted transfer to our hospital.

Upon admission, concomitant with hematological toxicity, severe liver injury was evident (March 25: ALT 474 U/L, AST 369 U/L; March 27: ALT peaked at 738 U/L, AST 369 U/L, ALP 42 U/L, GGT 138 U/L, TBil 16.6 umol/L, DBil 11.3 umol/L, albumin 26.6 g/L, INR 1.1). Based on ALT and ALP upper limits of normal of 40 U/L and 150 U/L, respectively, the R ratio was approximately 65.9, consistent with a hepatocellular pattern of liver injury. Blood cultures drawn on admission later grew methicillin-resistant coagulase-negative staphylococci (MRCNS) (samples sent March 24, reported positive in aerobic bottles on March 27; anaerobic bottles reported positive on March 31), and sputum culture yielded *Pseudomonas aeruginosa* (sample sent March 24, reported positive on March 27). The patient had infection but did not meet sepsis criteria.

Antifungal therapy was switched to isavuconazonium sulfate capsules from March 25 to March 27, but was stopped because of concern about liver function. The patient subsequently received nebulized amphotericin B liposome from March 28 to March 30, followed by intravenous amphotericin B cholesteryl sulfate complex from March 31 to April 8. Based on susceptibility results, antibacterial therapy was adjusted to piperacillin-tazobactam, vancomycin (for MRCNS), and later meropenem. Hepatoprotective therapy (magnesium isoglycyrrhizinate, glutathione, bicyclol) and multiple doses of G-CSF were administered.

**FIGURE 1:**
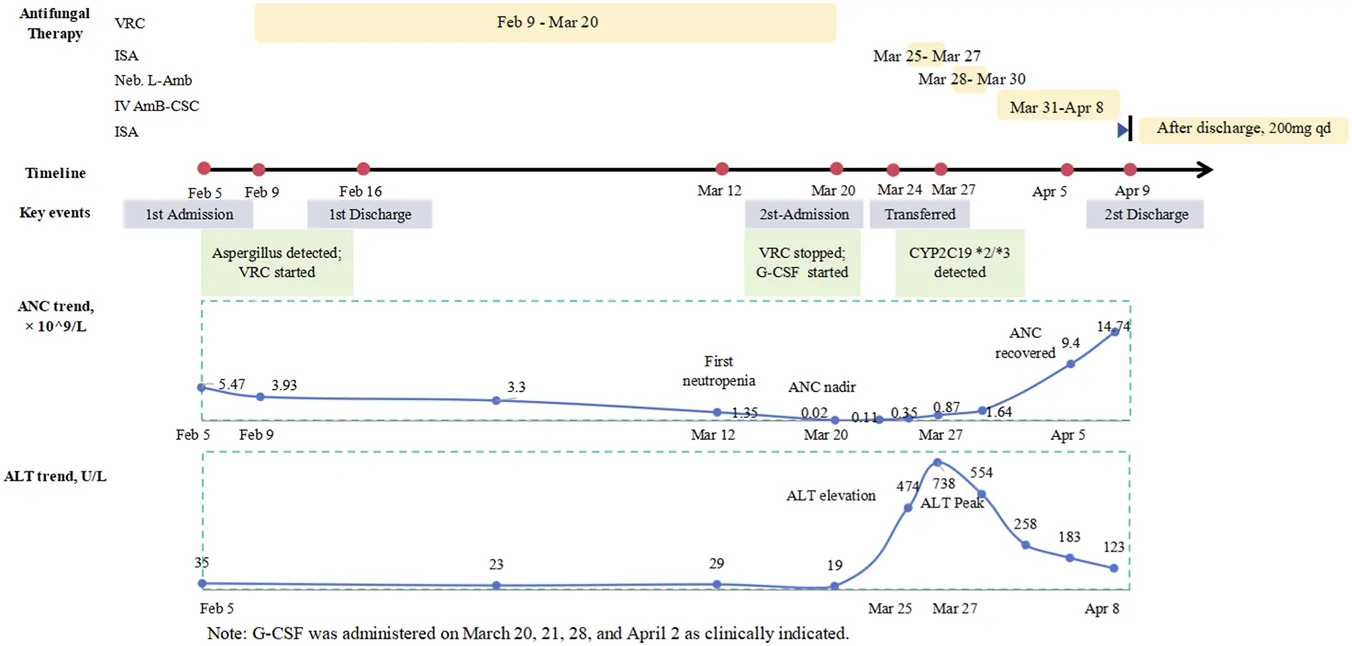
Patient treatment timeline. VRC, voriconazole; ISA, isavuconazonium; Neb., Nebulized; L-Amb, liposomal amphotericin B; IV, intravenous; AmB-CSC, amphotericin B cholesteryl sulfate complex; G-CSF, granulocyte colony-stimulating factor; ANC, absolute neutrophil count; ALT, alanine aminotransferase.

The patient’s fever subsided on April 4, coinciding with neutrophil recovery after multiple G-CSF administrations. At discharge on April 9, WBC was 20.43 × 10^9^/L, ANC 14.74 × 10^9^/L, ALT 123 U/L, AST 84 U/L, ALP 41 U/L, GGT 194 U/L, TBil 8.2 umol/L, DBil 3.8 umol/L, Alb 28.1 g/L, INR 1.01, CRP 14.1 mg/L, and PCT 0.08 ng/mL. The patient was discharged in stable condition with isavuconazonium sulfate capsules 200 mg qd after discharge. After discharge, the patient reported feeling well but did not undergo follow-up laboratory testing or imaging; therefore, objective extended post-discharge outcome data are not available. A treatment timeline is summarized in Figure 1.

## Discussion

This report presents a compelling case of concurrent, severe life-threatening toxicity—profound neutropenia and significant hepatocellular injury—in a patient receiving voriconazole, which we analyze in the context of his CYP2C19 PM status. The case illuminates critical gaps in clinical vigilance and provides a framework for personalized risk mitigation.

The causality of voriconazole-associated neutropenia was evaluated through systematic analysis of the clinical timeline and competing explanations. Neutropenia (ANC 1.35 × 10^9^/L) was detected on March 12 after approximately 30 days of voriconazole exposure, progressing to its nadir (ANC 0.02 × 10^9^/L) by March 20 while the patient was still receiving voriconazole. The patient had no documented underlying hematological disorder. Although short courses of ceftizoxime and moxifloxacin were used early in the course, these agents were less temporally consistent with the profound ANC nadir. Fever and subsequent MRCNS bacteremia occurred after neutropenia was established, suggesting infection secondary to pre-existing neutropenia; however, infection and systemic inflammation remain important clinical confounders. The Naranjo scale score (Naranjo et al., 1992) remained in the probable range (5 points) (Supplementary Table S1). The *CYP2C19* PM genotype provides a plausible pharmacological rationale for presumed voriconazole accumulation, but this mechanism cannot be confirmed without TDM.

For the hepatocellular injury, application of the revised RUCAM scale (Danan and Benichou, 1993; Andrade et al., 2019) yielded a probable causality score of six points (Supplementary Table S2). This assessment incorporated latency, dechallenge, age and alcohol history, concomitant medications, alternative-cause assessment, previous evidence of voriconazole hepatotoxicity, and absence of rechallenge. The dechallenge was rapid: ALT decreased from a peak of 738 U/L on 27 March 2025 to 258 U/L on 2 April 2025—a 65% reduction within 6 days of the peak, supporting the highest dechallenge score. The score reflects conservative re-scoring, including 0 points for alcohol use (the patient had abstained since early February) and −2 points for concomitant medications, recognizing that isavuconazonium sulfate, a triazole antifungal and a known hepatotoxin, was initiated on 25 March 2025 (when ALT was already 474 U/L) and discontinued on March 27 because of worsening liver function—a 2-day time-to-onset fully compatible with hepatotoxicity. While the clinical likelihood of isavuconazole contribution is judged lower than that of voriconazole, formal RUCAM scoring requires −2 for this criterion. Alternative causes were assessed with viral hepatitis serology, EBV/CMV plasma DNA PCR, liver-related autoantibodies, abdominal CT, and abdominal vascular ultrasound. Anti-HAV IgM, HBsAg, anti-HBc IgM, anti-HCV, and HEV IgM were negative; HBV DNA and HCV RNA quantitative PCR assays were not performed; HEV RNA was not performed; EBV DNA and CMV DNA were negative; and imaging showed multiple hepatobiliary cysts without biliary obstruction or vascular evidence of ischemic injury. We systematically evaluated all concomitant medications. Vancomycin and beta-lactam antibiotics, including piperacillin-tazobactam, meropenem, and cephalosporins, have been associated with rare neutropenia and/or liver enzyme abnormalities, and fluoroquinolones such as moxifloxacin can rarely cause liver injury. However, ceftizoxime and moxifloxacin were used in early February and discontinued well before the ANC decline; ceftazidime was initiated on the day the ANC nadir was documented; and piperacillin-tazobactam, vancomycin, and meropenem were started around or after the ALT peak. Therefore, these drugs were treated as potential confounders, but their temporal relationships were less consistent with the initial neutropenia and hepatocellular injury than the prolonged voriconazole exposure.

In this specific patient, the *CYP2C19* *2/*3 genotype represents the most plausible mechanistic explanation for presumed excessive drug exposure, though direct confirmation *via* TDM was not available. As a PM, his capacity to hydroxylate voriconazole is expected to be severely impaired, a phenotype that would be anticipated to result in pronounced and sustained drug accumulation based on established pharmacokinetic principles. However, we acknowledge that voriconazole-associated liver injury is a complex, multifactorial phenomenon. A recent large cohort study by Lou et al. (2025) demonstrated that several independent risk factors for voriconazole-induced liver injury, including intravenous administration, pre-existing liver cirrhosis, diabetes mellitus, and elevated baseline levels of urea, triglycerides, and C-Reactive Protein. Furthermore, voriconazole carries a high DILIrank score (Chen et al., 2016), indicating a substantial intrinsic risk for hepatotoxicity even in patients without pharmacogenetic risk factors, with proposed mechanisms including oxidative stress (Du et al., 2025). Indeed, case reports of severe hepatotoxicity in patients with normal CYP2C19 metabolism exist (Gu et al., 2023). In our patient, we hypothesize that the PM genotype may have acted as a potent effect modifier. It most likely amplified the intrinsic, dose-dependent hepatotoxic potential of the drug by predisposing to a state of sustained supratherapeutic exposure, potentially overwhelming the detoxification capacity of hepatocytes in a vulnerable elderly patient. Thus, we view the genotype not as the sole or proven cause, but as a critical predisposing factor that most likely contributed to severe, life-threatening toxicity in this individual. We emphasize that this interpretation remains inferential in the absence of measured plasma voriconazole concentrations.

Furthermore, it is critical to recognize that a ‘PM phenotype’ can be acquired even in patients with a normal genotype (extensive or intermediate metabolizers) through a process called phenoconversion. This can be caused by moderate-to-strong CYP2C19 inhibitors, such as omeprazole or fluconazole, which may be co-prescribed. This case, while involving a genotypic PM, therefore highlights a broader principle: clinicians must be vigilant for both genetic and drug-induced (phenocopied) impairments in metabolism. A patient with a normal genotype on a potent CYP2C19 inhibitor is functionally equivalent to a genotypic PM and carries a similar risk of accumulation and toxicity.

The mechanisms linking presumed excessive exposure to toxicity remain incompletely understood. Hepatotoxicity is thought to involve mitochondrial injury and oxidative stress (Haegler et al., 2017). The pathogenesis of agranulocytosis is less clear. In this patient, no bone marrow examination, voriconazole plasma concentrations, or mechanistic biomarkers were available. Therefore, a direct inhibitory effect of high voriconazole exposure on myeloid progenitor cells should be considered a hypothesis rather than a demonstrated mechanism in this case (Johnston et al., 2015). The co-occurrence of neutropenia and hepatocellular injury may reflect a shared vulnerability to presumed elevated voriconazole exposure, but this interpretation remains inferential.

For clinicians, this case reinforces a stratified management approach for patients requiring long-term voriconazole: 1) pre-therapy risk stratification, including consideration of *CYP2C19* genotyping in high-risk populations; 2) genotype-informed dosing or selection of an alternative antifungal when a poor metabolizer genotype is identified; 3) early TDM, ideally by day 5–7 of therapy, to maintain trough concentrations within the recommended therapeutic window; 4) multi-organ monitoring, including complete blood counts as well as liver function tests, especially during the first 4–6 weeks of therapy; and 5) prompt toxicity response, including discontinuation or switching of the suspected drug and targeted supportive care such as G-CSF for severe neutropenia. Clinical pharmacists can play a key role in interpreting genetic and TDM results, identifying adverse reactions, and promoting pharmacovigilance for high-risk drugs such as voriconazole.

This case has several limitations. The most significant limitation is the absence of voriconazole TDM data, which precludes a definitive correlation between the *CYP2C19* PM genotype, predicted elevated drug concentrations, and observed toxicities. Without measured plasma levels, we cannot definitively conclude that drug accumulation was the primary driver of toxicity. The interpretation is also limited by the complex clinical course, concomitant antibacterial and antifungal exposure, infection and systemic inflammation, absence of bone marrow examination, and absence of mechanistic biomarkers for neutropenia. Although alternative causes of liver injury were systematically investigated, some tests were not performed: HEV RNA was not available, and quantitative HBV DNA and HCV RNA PCR were not pursued because HBsAg and anti-HCV were negative. Given the negative HEV IgM, the absence of HEV-risk exposures, and the clinical course inconsistent with acute viral hepatitis, HEV infection is an unlikely confounder; similarly, the negative serological screening makes occult hepatitis virus infection improbable. In addition, after discharge the patient reported feeling well but did not undergo follow-up laboratory or imaging examinations, limiting objective assessment of long-term outcomes. Rather than claiming a definitive causal chain, we present this case as a high-probability risk scenario in which the convergence of genotype, temporal sequence, dechallenge, and structured causality assessment makes presumed voriconazole accumulation the most plausible explanation while acknowledging residual uncertainty.

## Conclusion

In this case report, a patient receiving voriconazole developed life-threatening neutropenia and hepatocellular liver injury. The convergence of *CYP2C19* PM genotype, temporal sequence, and structured causality assessment supports presumed voriconazole accumulation as the most plausible unifying explanation, although definitive attribution is precluded by the absence of TDM data and by competing clinical factors. This case underscores that clinical vigilance during voriconazole therapy should extend beyond hepatotoxicity to include serious hematological suppression. Pharmacogenomic assessment, early TDM, and systematic multi-organ surveillance may help personalize therapy, anticipate toxicity, and improve the safety of patients requiring prolonged voriconazole treatment.

## Data Availability

The original contributions presented in the study are included in the article/Supplementary Material, further inquiries can be directed to the corresponding author.
