# Supplementary material for: Case Report: Dual toxicity of voriconazole in a CYP2C19 poor metabolizer: a case analysis of life-threatening neutropenia and hepatocellular injury
Source: Front Pharmacol. 2026 Aug 21;17:1875506. doi: 10.3389/fphar.2026.1875506 (PMC13541743; doi:10.3389/fphar.2026.1875506)
Supplement: Supplementary file 1 [file Table1.docx]

**Supplementary Table S1. Naranjo scores for voriconazole-induced neutropenia with fever**

| **Question** | **Yes** | **No** | **Don't know** | **Score** | **Rationale** |
| --- | --- | --- | --- | --- | --- |
| 1. Are there previous conclusive reports on this reaction? | +1 | 0 | 0 | 1 | Neutropenia/agranulocytosis is listed in voriconazole prescribing information and has been reported with azole therapy. |
| 2. Did the adverse event appear after the suspected drug was given? | +2 | -1 | 0 | 2 | Neutropenia appeared approximately 30 days after voriconazole initiation. |
| 3. Did the adverse reaction improve when the drug was discontinued or a specific antagonist was administered? | +1 | 0 | 0 | 1 | ANC recovered after voriconazole discontinuation and G-CSF/supportive therapy. |
| 4. Did the adverse reaction reappear when the drug was readministered? | +2 | -1 | 0 | 0 | No formal rechallenge was performed. |
| 5. Are there alternative causes that could have caused the reaction? | -1 | +2 | 0 | 0 | Alternative causes (infection, other anti-infectives) exist but are temporally less consistent; whether they could alone have caused the reaction cannot be definitively determined. |
| 6. Did the reaction reappear when a placebo was given? | -1 | +1 | 0 | 0 | No placebo was administered. |
| 7. Was the drug detected in any body fluid in toxic concentrations? | +1 | 0 | 0 | 0 | Voriconazole plasma concentration was not available. |
| 8. Was the reaction more severe when the dose was increased, or less severe when the dose was decreased? | +1 | 0 | 0 | 0 | No dose escalation or reduction was performed. |
| 9. Did the patient have a similar reaction to the same or similar drugs in any previous exposure? | +1 | 0 | 0 | 0 | No prior similar exposure or reaction was documented. |
| 10. Was the adverse event confirmed by objective evidence? | +1 | 0 | 0 | 1 | Serial CBC confirmed profound neutropenia with ANC nadir 0.02 x 10^9/L. |
| **Total score** |  |  |  | **5** | **Probable adverse drug reaction.** |
| Note: Total score >= 9: definite; 5-8: probable; 1-4: possible; <= 0: doubtful. ANC: absolute neutrophil count; CBC: complete blood count; G-CSF, granulocyte colony-stimulating factor. | | | | | |
