# Supplementary material for: Case Report: Dual toxicity of voriconazole in a CYP2C19 poor metabolizer: a case analysis of life-threatening neutropenia and hepatocellular injury
Source: Front Pharmacol. 2026 Aug 21;17:1875506. doi: 10.3389/fphar.2026.1875506 (PMC13541743; doi:10.3389/fphar.2026.1875506)
Supplement: Supplementary file 2 [file Table2.docx]

**Supplementary Table S2. RUCAM Scale for Voriconazole-Induced Hepatocellular Drug Induced Liver Injury**

| **Items for Hepatocellular Injury** | **Score** | **Result** | **Rationale** |
| --- | --- | --- | --- |
| 1. Time to onset from the beginning of the drug/herb |  |  | Voriconazole began on February 9, 2025. Marked ALT elevation was identified on March 25 and peaked on March 27, within the compatible 5-90 day window. |
| ● 5–90 days (rechallenge:1–15 days) | +2 | +2 |  |
| ● <5 or >90 days (rechallenge:>15 days) | +1 |  |  |
| Alternative:Time to onset from cessation of the drug/herb |  |  |  |
| ● ≤15 days (except for slowly metabolized chemicals: >15 days) | +1 |  |  |
| 2. Course of ALT after cessation of the drug/herb |  |  | ALT peaked at 738 U/L on March 27 and decreased to 258 U/L on April 2 |
| Percentage difference between ALT peak and N |  |  |  |
| ● Decrease ≥ 50% within 8 days | +3 | +3 |  |
| ● Decrease ≥ 50% within 30 days | +2 |  |  |
| ● No information or continued drug use | 0 |  |  |
| ● Decrease ≥ 50% after the 30th day | 0 |  |  |
| ● Decrease < 50% after the 30th day or recurrent increase | −2 |  |  |
| 3. Risk factors |  |  | The patient had a history of alcohol consumption of approximately 50 mL of liquor per day, but had abstained since early February 2025.  The patient was 71 years old. |
| ● Alcohol use (current drinks/d: > 2 for women, >3 for men) | +1 |  |  |
| ● Alcohol use (current drinks/d:≤ 2 for women, ≤3 for men) | 0 | +0 |  |
| ● Age ≥ 55 years | +1 | +1 |  |
| ● Age < 55 years | 0 |  |  |
| 4. Concomitant drug(s)/herb(s) |  |  | The clinical course included ceftizoxime, moxifloxacin, ceftazidime, piperacillin-tazobactam, vancomycin, meropenem, isavuconazonium sulfate, and amphotericin B formulations; these exposures were treated as potential confounders. |
| ● None or no information | 0 |  |  |
| ● Concomitant drug/herb with incompatible time to onset | 0 |  |  |
| ● Concomitant drug/herb with compatible or suggestive time to onset | −1 |  |  |
| ● Concomitant drug/herb known as hepatotoxin and with compatible or suggestive time to onset delete marking right side above | −2 | -2 |  |
| ● Concomitant drug/herb with evidence for its role in this case (positive rechallenge or validated test) | −3 |  |  |
| 5. Search for alternative causes | Tick if negative | Tick if not done | Anti-HAV IgM, HBsAg, anti-HBc IgM, anti-HCV, and HEV IgM were negative. Dedicated quantitative HBV DNA and HCV RNA PCR assays were not performed. HEV RNA was not performed. EBV DNA and CMV DNA PCR were negative. Liver-related autoantibodies were negative. Abdominal CT and vascular ultrasound showed multiple hepatobiliary cysts without biliary obstruction or vascular evidence of ischemic injury. The patient had infection but did not meet sepsis criteria. |
| Group I (7 causes) |  |  |  |
| ● HAV: Anti-HAV-IgM | √ |  |  |
| ● Hepatobiliary sonography / colour Doppler | √ |  |  |
| ● HCV:Anti-HCV,HCV-RNA |  | √ |  |
| ● HEV:Anti-HEV-IgM, anti-HEV-IgG, HEV-RNA |  | √ |  |
| ● Hepatobiliary sonography/colour Doppler sonography of liver vessels/endosonography/CT/MRC | √ |  |  |
| ● Alcoholism (AST/ALT ≥ 2) | √ |  |  |
| ● Acute recent hypotension history (particularly if underlying heart disease) | √ |  |  |
| Group II (5 causes) |  |  |  |
| ● Complications of underlying disease(s) such as sepsis,metastatic malignancy,autoimmune hepatitis,chronic hepatitis B or C,primary biliary cholangitis or sclerosing cholangitis,genetic liver diseases | √ |  |  |
| ● Infection suggested by PCR and titer change for |  |  |  |
| ● CMV (anti-CMV-IgM,anti-CMV-IgG) | √ |  |  |
| ● EBV (anti-EBV-IgM,anti-EBV-IgG) | √ |  |  |
| ● HSV (anti-HSV-IgM,anti-HSV-IgG) |  | √ |  |
| ● VZV (anti-VZV-IgM,anti-VZV-IgG) |  | √ |  |
| Evaluation of groups I and II |  |  |  |
| ● All causes-groups I and II—reasonably ruled out | +2 |  |  |
| ● The 7 causes of group I ruled out | +1 |  |  |
| ● 6 or 5 causes of group I ruled out | 0 | +0 |  |
| ● Less than 5 causes of group I ruled out | -2 |  |  |
| ● Alternative cause highly probable | -3 |  |  |
| 6. Previous hepatotoxicity of the drug/herb |  |  | Elevated transaminases and hepatotoxicity are recognized in voriconazole product information and literature. |
| ● Reaction labelled in the product characteristics | +2 | +2 |  |
| ● Reaction published but unlabelled | +1 |  |  |
| ● Reaction unknown | 0 |  |  |
| 7. Response to unintentional reexposure |  |  | No voriconazole rechallenge was performed. |
| ● Doubling of ALT with the drug/herb alone,provided ALT below 5N before reexposure | +3 |  |  |
| ● Doubling of ALT with the drug(s)/herb(s) already given at the time of first reaction | +1 |  |  |
| ● Increase of ALT but less than N in the same conditions as for the first administration | −2 |  |  |
| ● Other situations | 0 | +0 |  |
| Total score for the case | | 6 |  |
| Note: Total score and resulting causality grading: ≤ 0, excluded; 1–2, unlikely; 3–5, possible; 6–8, probable; and ≥ 9, highly probable. Abbreviations: ALT, alanine aminotransferase; AST, aspartate aminotransferase; CMV, cytomegalovirus; CT, computer tomography; DILI, drug induced liver injury; EBV, epstein - barr virus; HAV, hepatitis A virus; HBc, hepatitis B core; HBsAg, hepatitis B antigen; HBV, hepatitis B virus; HCV, hepatitis C virus; HEV, hepatitis E virus; HILI, herb induced liver injury; HSV, herpes simplex virus; MRC, magnetic resonance cholangiography; N, upper limit of the normal range; RUCAM, Roussel Uclaf Causality Assessment Method; VZV, varicella zoster virus. | | | |
