# Supplementary material for: Case Report: Dual toxicity of voriconazole in a CYP2C19 poor metabolizer: a case analysis of life-threatening neutropenia and hepatocellular injury
Source: Front Pharmacol. 2026 Aug 21;17:1875506. doi: 10.3389/fphar.2026.1875506 (PMC13541743; doi:10.3389/fphar.2026.1875506)
Supplement: Supplementary file 3 [file Table3.docx]

**Supplementary Table S3. Detailed medication orders and therapeutic interventions during the clinical course**

| **Category** | **Drug / intervention** | **Dose / route / frequency** | **Start date** | **End date** | **Clinical context** |
| --- | --- | --- | --- | --- | --- |
| Antifungal | Voriconazole tablets | 400 mg q12h po for loading on Feb 9; then 200 mg q12h po | 2025-02-09 | 2025-03-20 | Initial treatment for invasive pulmonary aspergillosis; discontinued after severe neutropenia and liver injury were recognized. |
|  | Isavuconazonium sulfate capsules | 200 mg q8h po | 2025-03-25 | 2025-03-26 | Alternative azole after voriconazole discontinuation. |
|  | Isavuconazonium sulfate capsules | 200 mg qd po | 2025-03-27 | 2025-03-27 | Stopped because of concern about liver function. |
|  | Amphotericin B liposome for injection | 10 mg + sterile water for injection 10 mL, bid nebulization | 2025-03-28 | 2025-03-30 | Nebulized/local antifungal therapy after azole discontinuation. |
|  | Amphotericin B cholesteryl sulfate complex for injection | 50 mg + 5% glucose injection 250 mL IV pump | 2025-03-31 | 2025-03-31 | Systemic amphotericin B formulation; dose escalation phase. |
|  | Amphotericin B cholesteryl sulfate complex for injection | 100 mg + 5% glucose injection 250 mL IV pump | 2025-04-01 | 2025-04-01 | Systemic amphotericin B formulation; dose escalation phase. |
|  | Amphotericin B cholesteryl sulfate complex for injection | 150 mg + 5% glucose injection 250 mL qd IV pump | 2025-04-02 | 2025-04-08 | Systemic antifungal treatment before discharge. |
| Post-discharge antifungal | Isavuconazonium sulfate capsules | 200 mg qd po | 2025-04-09 | 2025-04-15 | Continued oral antifungal therapy after discharge; patient reported feeling well but did not undergo follow-up testing or imaging. |
| Antibacterial / antiviral | Moxifloxacin hydrochloride and sodium chloride injection | 0.4 g qd ivgtt | 2025-02-05 | 2025-02-16 | Early antibacterial therapy. |
|  | Ceftizoxime sodium for injection | 2 g + 0.9% sodium chloride injection 50 mL q12h ivgtt | 2025-02-06 | 2025-02-11 | Early antibacterial therapy. |
|  | Oseltamivir phosphate capsules | 75 mg bid po | 2025-02-07 | 2025-02-16 | Antiviral therapy during early admission. |
|  | Ceftazidime for injection | 2 g + 0.9% sodium chloride injection 100 mL q12h ivgtt | 2025-03-20 | 2025-03-24 | Empiric antibacterial therapy after fever and severe neutropenia. |
|  | Piperacillin sodium and tazobactam sodium for injection | 4.5 g + 0.9% sodium chloride injection 100 mL q12h ivgtt | 2025-03-25 | 2025-03-27 | Adjusted antibacterial therapy after transfer. |
|  | Piperacillin sodium and tazobactam sodium for injection | 4.5 g + 0.9% sodium chloride injection 100 mL q8h ivgtt | 2025-03-28 | 2025-04-01 | Continued antibacterial therapy based on clinical course/susceptibility. |
|  | Vancomycin hydrochloride for injection | 1 g + 0.9% sodium chloride injection 250 mL stat ivgtt | 2025-03-31 | 2025-03-31 | Coverage for MRCNS bloodstream infection. |
|  | Vancomycin hydrochloride for injection | 350 mg + 0.9% sodium chloride injection 250 mL q12h ivgtt | 2025-04-01 | 2025-04-08 | Continued coverage for MRCNS. |
|  | Meropenem for injection | 1 g + 0.9% sodium chloride injection 100 mL q8h ivgtt | 2025-04-02 | 2025-04-08 | Escalated antibacterial therapy. |
| Supportive / hepatoprotective | Human granulocyte colony-stimulating factor injection | 100-150 ug per dose, subcutaneous/injection as clinically indicated | 2025-03-20 | 2025-04-04 | Supportive therapy for severe neutropenia; multiple administrations. |
|  | Silibinin capsules | 105 mg tid po | 2025-02-10 | 2025-02-25 | Hepatoprotective/supportive therapy during early course. |
|  | Magnesium isoglycyrrhizinate injection | 200 mg + 0.9% sodium chloride injection 250 mL qd ivgtt | 2025-03-25 | 2025-04-08 | Hepatoprotective therapy during DILI management. |
|  | Glutathione for injection | 1.8 g + 0.9% sodium chloride injection 250 mL qd ivgtt | 2025-03-25 | 2025-04-08 | Hepatoprotective therapy during DILI management. |
|  | Bicyclol tablets | 25 mg bid po | 2025-03-26 | 2025-04-08 | Hepatoprotective therapy during DILI management. |
| Note: qd, once daily; bid, twice daily; tid: three times daily;; q8h/q12h, every 8/12 hours; DILI, drug-induced liver injury; G-CSF, granulocyte colony-stimulating factor; IPA, invasive pulmonary aspergillosis; po, orally; ivgtt, intravenous drip; MRCNS, methicillin-resistant coagulase-negative staphylococci. | | | | | |
